# The In Vitro Antioxidant and Immunomodulatory Effects of the Irish Monofloral Ivy and Heather Honey Varieties †

**DOI:** 10.3390/ijms26083625

**Published:** 2025-04-11

**Authors:** Emma Browne, Siobhán Kavanagh, Sinead Devery

**Affiliations:** 1 Bioscience Research Institute, Technological University of the Shannon, Athlone, Co., N37HD68 Westmeath, Ireland; 2 Department of Pharmaceutical Sciences and Biotechnology, Technological University of the Shannon, Athlone, Co., N37HD68 Westmeath, Ireland

**Keywords:** Irish honey, monofloral honey, heather honey, ivy honey, THP-1, antioxidant activity, immunomodulation, cytokine regulation, oxidative stress

## Abstract

Honey has long been valued for its medicinal properties, yet the therapeutic potential of Irish monofloral honey remains largely unexplored. This study investigates the antioxidant and immunomodulatory effects of Irish ivy (*Hedera helix*) and heather (*Calluna vulgaris*) honey samples on PMA-differentiated THP-1 macrophages, a well-characterised immune model. Antioxidant capacity was assessed through free radical scavenging assays, DPPH and ORAC, while qPCR analysis examined the key inflammatory markers. Both the heather and ivy honey varieties demonstrated antioxidant activity, with heather honey exhibiting the highest total phenolic content (TPC), and ivy honey stimulating Nrf2 activation. Manuka honey showed the strongest radical scavenging capacity, as reflected in its higher ORAC and DPPH values. These findings suggest that the different honey varieties may exert antioxidant effects through distinct mechanisms. Exposure to honey reduced oxidative stress and upregulated the expression of a key antioxidant transcription regulator (Nrf2) and an associated downstream antioxidant defence enzyme, superoxide dismutase (SOD). Additionally, both the honey types exhibited immunomodulatory effects, upregulating pro-inflammatory cytokines, such as TNF-α and IL-1β, while increasing the expression of the anti-inflammatory cytokine IL-10. These findings suggest potential bioactive properties that warrant further investigation. Given the growing interest in alternative treatments for inflammation-related conditions, further research is warranted to determine whether the observed in vitro effects translate into clinically relevant outcomes. This study expands the current understanding of Irish monofloral honey, reinforcing its potential as a functional bioactive compound with relevance in antioxidant therapies, immune modulation, and wound healing.

## 1. Introduction

Honey has long been recognised for its medicinal properties, offering antimicrobial [1,2], anti-inflammatory [3], antioxidant [4], and wound-healing benefits [5,6,7]. While much research has focused on manuka honey and its methylglyoxal (MGO)-driven antibacterial activity [8], other monofloral honey varieties remain significantly understudied. Recent investigations have highlighted the bioactive potential of Irish monofloral honey, particularly heather (Calluna vulgaris) [9,10] and ivy (Hedera helix) honey [9], which exhibit high total phenolic contents (TPC), potentially correlating to strong antioxidant and other therapeutic effects (immunomodulatory and antimicrobial [11,12,13]). While manuka honey has been extensively studied for its medicinal properties, the therapeutic mechanisms of Irish honey varieties remain largely unexplored. Given their distinct botanical origins and phytochemical compositions, investigating how Irish ivy and heather honey influence antioxidant pathways and immune modulation is essential to understanding their potential functional applications.

The antioxidant capacity of honey is critical in mitigating oxidative stress, which plays a central role in inflammation, tissue damage, and chronic disease [14,15]. Many of honey’s bioactive effects are linked to its rich composition of phenolic compounds, flavonoids, and enzymatic components, which contribute to its free radical scavenging ability and cellular protective mechanisms [13]. Preliminary studies suggest that heather and ivy honey have among the highest TPC values recorded for Irish honey varieties [9,10], yet their effects on antioxidant regulation and immune responses in human cellular models have yet to be fully explored.

The composition and therapeutic efficacy of a honey variety are influenced by multiple factors, including geographical region, floral source, and environmental stressors [13,16,17]. Other studies on heather honey from Scotland [18], Poland [19,20], Romania [21], and the Western Balkans [22] have consistently reported a high TPC and strong antioxidant activity, often surpassing those of other local honey varieties. Scottish heather honey, for instance, has demonstrated antimicrobial activity against *Staphylococcus aureus* and *Escherichia coli*, showing similar efficacy to medical grade manuka honey [23]. Portuguese heather plants [24] and honey [25] and Polish heather honey varieties [26] are particularly rich in flavonoids, such as quercetin and catechins, which contribute to their anti-inflammatory and oxidative stress-reducing properties.

Ivy honey has received comparatively little scientific attention, yet emerging research highlights its potential as an antibacterial and antibiofilm agent. Italian ivy honey has been found to inhibit biofilm formation in clinically relevant bacterial pathogens, such as *Pseudomonas aeruginosa* and *Staphylococcus aureus*, suggesting a role in infection control [27]. In Turkey, ivy honey extracts have been reported to exhibit potent antibacterial effects against *Helicobacter pylori* [28]. The variability in honey composition across regions underscores the importance of local studies, as environmental conditions (light exposure and wet/dry weather), soil composition, and climate significantly influence the bioactive compounds present in nectar-producing plants [29,30], and consequently in the honey derived from them.

Beyond its antimicrobial and antioxidant properties, honey is increasingly recognised for its ability to modulate immune responses [31]. Immunomodulation is a critical factor in inflammation regulation, with excessive or dysregulated inflammatory responses contributing to chronic disease progression [32]. Honey’s ability to influence immune cell activity has been attributed to its complex biochemical composition, including bee defensin-1 [33], major royal jelly proteins (MRJPs) [34,35], and arabinogalactans [36], which interact with immune cells to regulate cytokine expression.

Some studies on manuka honey have demonstrated its capacity to modulate nuclear factor kappa B (NF-κB) signalling, a transcription factor involved in regulating immune responses, leading to a reduction in pro-inflammatory cytokines, such as tumour necrosis factor-alpha (TNF-α) and interleukin-1 beta (IL-1β), while promoting the expression of anti-inflammatory mediators like interleukin-10 (IL-10) [37]. Given the high TPC of Irish heather and ivy honey, it is plausible that they exert similar or even distinct immunomodulatory effects. However, no identifiable studies to date have examined their impact on immune cell function, highlighting a significant gap in our understanding of their therapeutic potential.

The well-documented regional and botanical variability in honey composition and bioactivity, even among samples of the same floral origin [13,16,17], underscores the importance of investigating locally sourced honey varieties for potentially unique therapeutic properties. While manuka honey remains the benchmark for therapeutic use, Irish monofloral honeys—particularly ivy and heather—have been underexplored. The aim of this study was to assess and compare the in vitro antioxidant and immunomodulatory capacity of Irish ivy and heather honeys relative to manuka honey and to elucidate their potential mechanisms of action. More specifically by examining the effect of Irish monofloral honey varieties on free radical scavenging, inflammatory cytokine expression, and antioxidant enzyme activity, this study aims to establish their role as natural antioxidant and immunomodulatory agents. This study builds upon prior research optimising the differentiation of THP-1 monocytes into macrophages using phorbol 12-myristate 13-acetate (PMA) [38], providing a well-characterised model for assessing honey’s immunomodulatory effects.

Given the growing interest in natural therapeutics and the increasing need for alternative antimicrobials and synthetic anti-inflammatory drugs, this research contributes to the expanding body of literature on medicinal honey. Understanding the bioactive potential of Irish honey varieties will not only support their development as standardised medicinal products, but may also offer novel insights into their clinical applications for inflammation management and wound healing.

## 2. Results

The effects of Irish ivy and heather honey on antioxidant activity, cytotoxicity, and immunomodulation were evaluated in PMA-differentiated THP-1 M1 macrophages. The antioxidant capacity of each honey variety was first assessed through TPC, DPPH (2,2-Diphenyl-1-picrylhydrazyl) radical scavenging, and ORAC (Oxygen Radical Absorbance Capacity) assays to determine their free radical neutralisation potential. Cytotoxicity (resazurin assay) was then evaluated to ensure the honey treatments were non-toxic to the macrophages at biologically relevant concentrations. The immunomodulatory effects of each honey variety were examined by measuring the expression of key inflammatory markers, including NF-κB, TNF-α, IL-1β, and IL-10, to assess their potential influence on the M1 macrophages. Lastly, the antioxidant effects of the honey on the macrophages were assessed by evaluating the changes in Nrf2 transcriptional regulation and the expression of the antioxidant enzyme SOD. Together, these findings provide a comprehensive understanding of the antioxidant and immunomodulatory properties of ivy and heather honey, offering insights into their potential therapeutic applications.

### 2.1. Antioxidant Capacity of Irish Heather and Ivy Honey Varieties

#### 2.1.1. Total Phenolic Content

**Figure 1:**
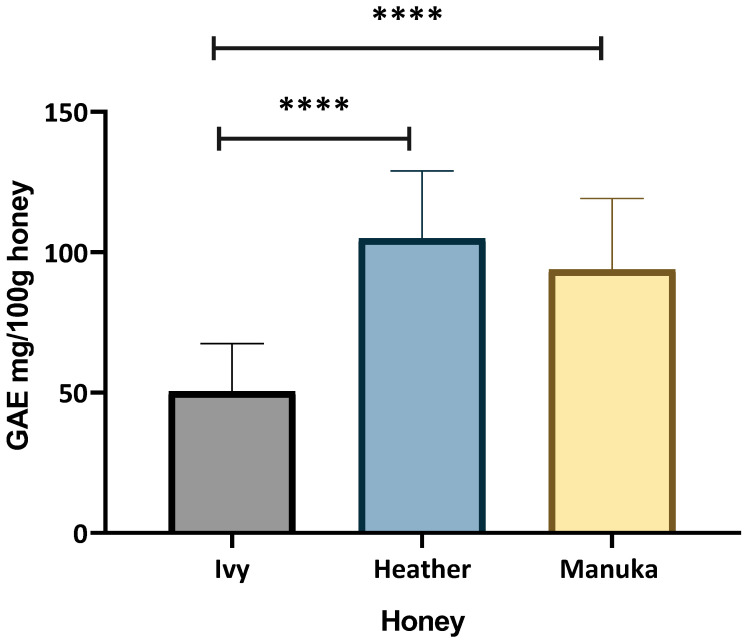
Average total phenolic content of ivy honey, heather honey, and manuka honey. Data represents mean TPC of individual confirmed monofloral honey varieties, with error bars representing standard deviation. Three technical replicates per sample with two individual experiments performed. TPC values were compared using one-way ANOVA with Bonferroni’s post-hoc multiple comparisons test. *p* < 0.0001 ****.

Figure 1 shows the mean TPC value for ivy honey (50.37 ± 16.67 Gallic Acid Equivalent (GAE) mg/100 g honey), heather honey (104.8 ± 28.866 GAE mg/100 g honey), and manuka honey (93.86 ± 25.28 GAE mg/100 g honey). Overall, the heather honey had a significantly higher TPC value compared to that of the ivy honey (*p* < 0.0001). Hence the findings from this study are similar to those of Kavangah et al. [9] who also reported a higher TPC for Irish heather honey (68.16 ± 2.73 GAE mg/100 g) versus that for Irish ivy honey (34 mg/100 g). However, in this study, the TPC for heather honey (104.8 ± 28.866 GAE/100 g honey) was markedly higher than that reported by Kavanagh et al. [9] and comparable to a study by Angioi et al. [10] for Irish heather honey (107 ± 4 mg GAE/100 g). This study also found the TPC of heather honey to be greater than that found for manuka honey (93.86 ± 25.28 mg GAE/100 g) (Figure 1). Overall, other studies that have assessed the TPC value of manuka honey reported a range of values between 52.8 mg/100 g by Hulea et al. [39], 56 mg/100 g by Deng et al. [40], and 105.08 mg/100 g by Ndungu et al. [41], with the highest reported TPC values for manuka honey being comparable to the TPC for the heather honey varieties in this study.

#### 2.1.2. Free Radical Scavenging

**Figure 2:**
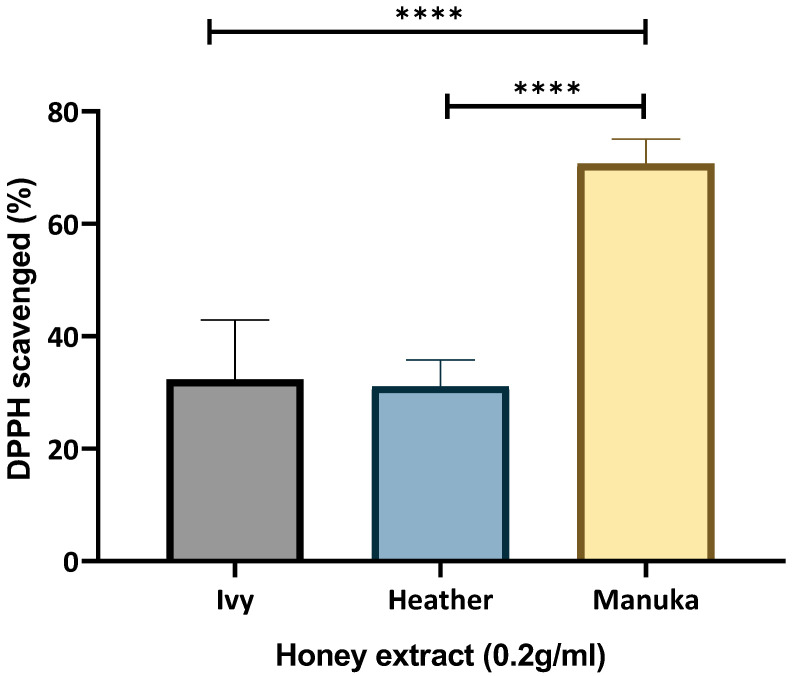
Free radical scavenging ability of honey using DPPH assay, depicting percent scavenged. Four technical replicates per sample with three individual experiments performed. Results analysed with one-way ANOVA and Bonferroni’s post-hoc test (**** *p* < 0.0001).

Both the ivy and heather honey demonstrated a comparable scavenging ability (32.336% ± 10.58 and 31.089% ± 4.724) in the DPPH assay (Figure 2). Manuka honey (MGO 250) demonstrated a scavenging ability value double that of either of the Irish honey sample (70.7185% ± 4.333) (Figure 2). Interestingly, heather honey previously showed a TPC value greater than that of manuka honey (104.84 ± 28.866 versus 93.86 ± 25.28 GAE mg/100 g honey, respectively) (Figure 1). Hence, the greater DPPH Radical Scavenging Ability (RSA) by manuka honey here may be attributed to the presence of additional bioactive compounds, such as MGO, unique flavonoid glycosides, vitamins (e.g., ascorbic acid), and enzymes that enhance its radical scavenging activity. Alternatively, the lower DPPH and ORAC values observed in heather honey, despite its higher measured TPC, may be attributed to the predominance of more hydrophilic phenolic compounds, which are less efficiently extracted into the methanolic phase used for analysis, subsequently affecting the DPPH and ORAC values. Future HPLC-PDA, LC-MS/MS, or NMR-based profiling is recommended to identify these constituents and validate their contribution to antioxidant capacity.

#### 2.1.3. Peroxyl Radical Neutralising (ORAC) Assay

**Figure 3:**
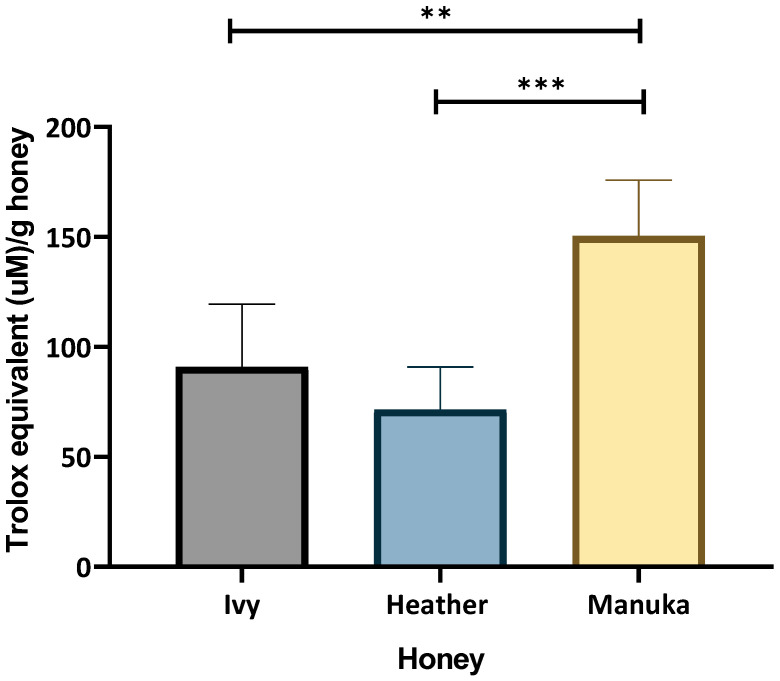
ORAC assay showing Trolox equivalent μM/g of honey to stabilise peroxyl radicals over time. Data are representative of four technical replicates per sample, with three individual experiments performed. Results analysed with one-way ANOVA and Bonferroni’s post-hoc test (** *p* < 0.01, *** *p* < 0.001).

The ORAC assay provides a time-based measure of how effectively a therapeutic can neutralise peroxyl radicals, capturing the extent and duration of antioxidant activity. The area under the curve was measured, and the Trolox equivalent (TE) was interpolated from a Trolox standard curve. It was determined that the ivy honey had a higher TE (90.83 ± 28.58 µM) per gram of honey compared to that of heather honey (71.464 ± 19.35 TE µM/g), with manuka honey exhibiting a significantly greater TE compared to those of both the ivy and heather honey samples (150.392 ± 25.48 TE µM/g), Figure 3.

### 2.2. The Immunomodulatory and Antioxidant Effects of Irish Honey Samples

To evaluate the immunomodulatory potential of Irish ivy and heather honey, the gene expression changes in the key inflammatory and antioxidant markers were assessed in the M1-polarised PMA-differentiated THP-1 macrophages. The expression of pro-inflammatory (NF-κB, TNF-α, and IL-1β), and anti-inflammatory (IL-10) biomarkers, the antioxidant transcription factor Nrf2, and the associated SOD enzyme were measured following the honey treatment using manuka as a standardised medicinal honey in addition to lab honey as a sugar control, accounting for the osmotic effects of honey. The expression of RPL37A and β-actin, as control genes, in the LPS-challenged macrophages were used to normalise for any changes in the gene expression of the pro- and anti-inflammatory biomarkers evaluated. Ultimately, we evaluated whether the Irish honey varieties exhibit immunomodulatory or antioxidant effects distinct from those of the other honey types.

#### 2.2.1. Cytotoxicity of Honey on Differentiated Macrophages (Resazurin Assay)

**Figure 4:**
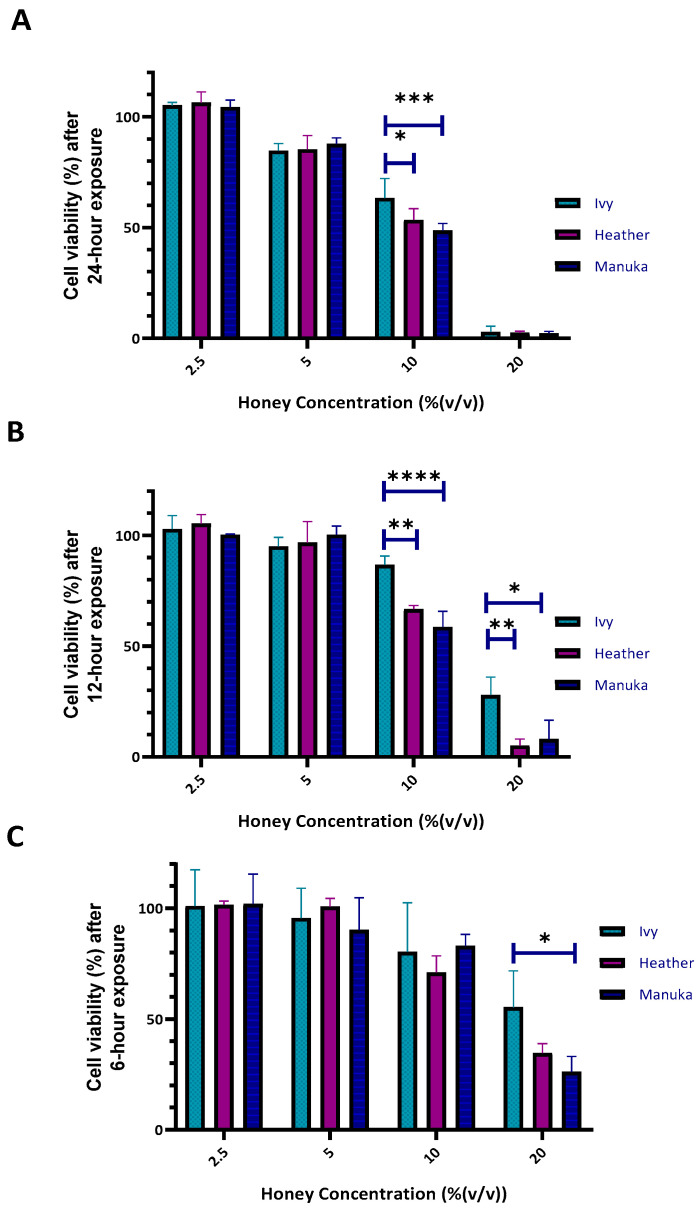
Cell viability (%) of differentiated THP-1 cells after (**A**) 24 h, (**B**) 12 h, and (**C**) 6 h exposure to honey at varying concentrations was determined using resazurin assay. Data shown as mean ± std. dev. from three independent experiments with six technical replicates. Data compared with two-way ANOVA and Bonferroni’s post hoc test (* *p* < 0.05, ** *p* < 0.01, *** *p* < 0.001, **** *p* < 0.0001).

A sub-cytotoxic concentration was determined for ivy, heather, and manuka honey exposure to the differentiated THP-1 cells for use in further downstream analysis to ensure that any observed biological effects are not confounded by cytotoxicity (inflammatory and antioxidant biomarker gene expression). The cells were exposed to varying concentrations (from 2.5% to 20% (*v*/*v*)) for three different exposure times (6, 12, and 24 h). A dose-dependent effect at all the three exposure times was observed for all the three honey samples (Irish ivy, Irish heather, and manuka honey), highlighting the need to identify a concentration that does not compromise cell viability. This approach ensures that any changes in gene expression observed in subsequent analyses are not confounded by cytotoxic effects, providing a clearer understanding of the honey varieties’ impact on cell health and functionality (Figure 4).

For the three honey samples, higher concentrations, (10% and 20% (*v*/*v*)) were cytotoxic for the differentiated THP-1 cells at 24 h exposure, with 10% (*v*/*v*) concentrations decreasing the cell viabilities for all the three honey samples tested (69.7% ± 2.5625 (std. dev.), 57.33% ± 0.698, and 54.1% ± 0.809 for ivy, heather, and manuka honey, respectively) (Figure 4A). Following 12 h exposure at 20% (*v*/*v*), a difference in cytotoxicity was observed for ivy honey (27.966% ± 8.11) in relation to heather (5.167% ± 2.913) and manuka 8.028% ± 8.525) (*p* < 0.01, and *p* < 0.05, respectively) (Figure 4B). A similar trend for THP-1 derived macrophage viability was also observed following 6 h exposure to all three honey samples at 20% (*v*/*v*), with ivy honey again exerting the lowest cytotoxic effect (55.34% ± 16.4) compared to those of both the heather (34.646% ± 4.233) and manuka (26.134% ± 6.952) honey (Figure 4C).

#### 2.2.2. The Immunomodulatory Effects of Irish Ivy and Heather Honey Samples on Inflammatory Marker Gene Expression

**Figure 5:**
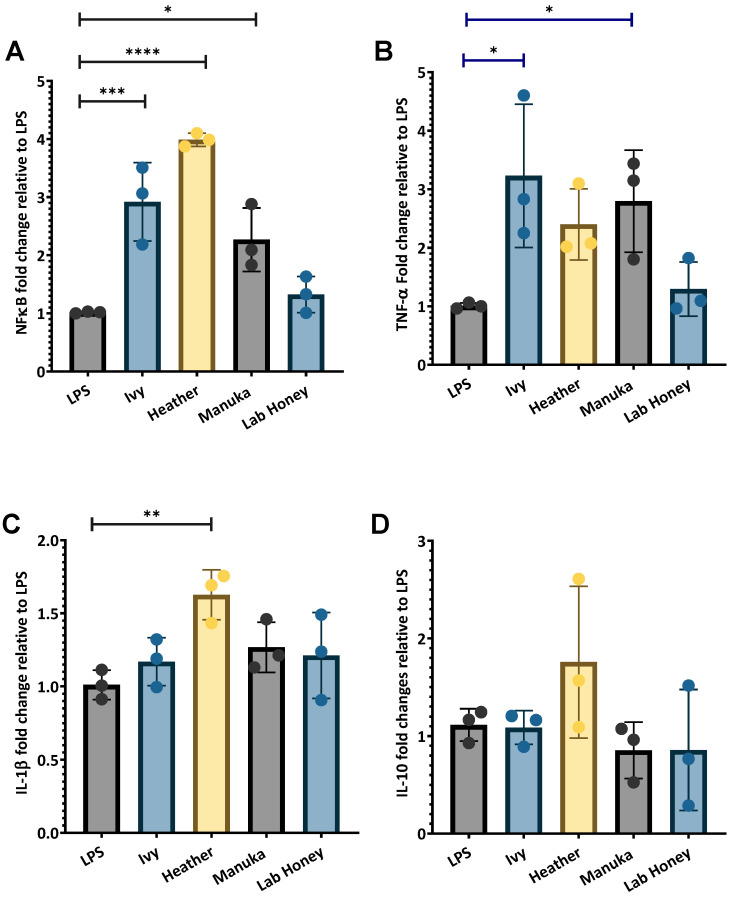
Key inflammatory gene expression in M1 polarised THP-1 cells after treatment with 2.5% (*v*/*v*) honey for 6 h. (**A**) NF-κB expression, (**B**) TNF-α expression, (**C**) IL-1β expression, and (**D**) IL-10 expression. Results are expressed as relative fold change in relation to control (LPS-treated THP-1 macrophages) and normalised to two reference genes (RPL37A and β-actin). Results are expressed as mean of three independent experiments, with error bars representing standard deviation. Results analysed with one-way ANOVA and Dunnett’s post-hoc test (* *p* < 0.05, ** *p* < 0.01, *** *p* < 0.001, **** *p* < 0.0001).

A significant increase in NF-κB expression was observed following 6 h exposure to 2.5% (*v*/*v*) for ivy (2.9-fold ± 0.6732, *p* < 0.001), heather (3.985-fold ± 0.11, *p* < 0.0001), and Manuka honey (2.268-fold ± 0.545, *p* < 0.05). A slight increase was observed with the lab honey (1.322-fold ± 0.31) (Figure 5A). TNF-α expression was also significantly upregulated by ivy honey (3.23-fold ± 1.225, *p* < 0.05) and manuka honey (2.795-fold ± 0.87, *p* < 0.05). Heather honey also increased TNF-α expression (2.397-fold ± 0.608), while the lab honey caused only a slight increase (1.294-fold ± 0.464) (Figure 5B). Among the tested honey varieties, heather honey demonstrated the greatest IL-1β induction (1.63-fold ± 0.17, *p* < 0.01) compared to those of manuka honey (1.267-fold ± 0.17), ivy honey (1.17-fold ± 0.16), and the lab honey (1.211-fold ± 0.29) (Figure 5C).

For IL-10, heather honey induced the highest expression (1.757-fold ± 0.78), indicating a greater effect on anti-inflammatory IL-10 regulation. In contrast, ivy honey resulted in a slight increase (1.086-fold ± 0.1713), suggesting a minimal impact on IL-10 expression. Manuka honey (0.852-fold ± 0.29) and the lab honey (0.856-fold ± 0.62) were associated with a decrease in IL-10 expression compared to that of the LPS-stimulated macrophage control (Figure 5D). These results suggest ivy honey primarily stimulates NF-κB and TNF-α, with a minimal influence on IL-1β or IL-10, while heather honey induces a more balanced immune response by stimulating NF-κB, TNF- α, IL-1β, and IL-10. The response to manuka honey mirrors that of ivy honey, while the lab honey had no major immunostimulatory effects.

#### 2.2.3. Response of Nrf2 Antioxidant Transcriptional Regulator and Downstream SOD to Honey Exposure

**Figure 6:**
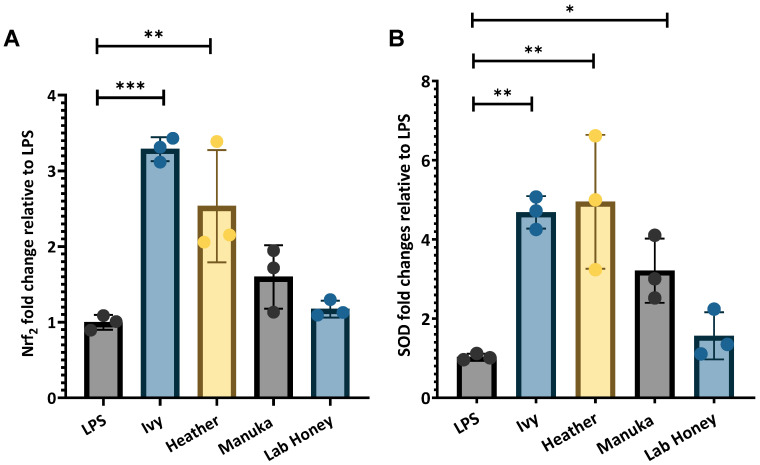
Antioxidant biomarkers expression in pro-inflammatory THP-1 macrophages after 6 h treatment with 2.5% (*v*/*v*) honey varieties. (**A**) Nrf2 expression. (**B**) SOD expression. Results expressed as relative fold change in relation to control (LPS-treated THP-1 macrophages, normalised to two reference genes RPL37A and β-actin). Data are representative of three independent experiments, and error bars represent standard deviation. For statistical analysis, we used one-way ANOVA with Dunnett’s post-hoc test (* *p* < 0.05, ** *p* < 0.01, *** *p* < 0.001).

Nrf2 and SOD gene expression was stimulated to a greater extent by ivy and heather honey than by manuka and the lab honey (Figure 6). Nrf2 expression was significantly upregulated in response to ivy honey (3.287-fold ± 0.159, *p* < 0.001) and heather honey (2.533-fold ± 0.743, *p* < 0.01) when normalised to reference gene (RPL37A and β-actin) expression in the LPS-stimulated M1 macrophage control. In contrast, manuka honey induced a moderate increase in Nrf2 expression (1.598-fold ± 0.42), while the lab honey resulted in minimal changes (1.173-fold ± 0.109) (Figure 6A).

SOD expression followed a similar trend, with both ivy honey (4.68-fold ± 1.69, *p* < 0.01) and heather honey (4.95-fold ± 1.69, *p* < 0.01) showing the highest levels of upregulation compared to that of the LPS-stimulated macrophage control. Manuka honey also increased SOD expression significantly (2.521-fold ± 0.811, *p* < 0.05), with the lab honey inducing only a slight increase (1.565-fold ± 0.596) (Figure 6B).

These results suggest that ivy and heather honey enhance antioxidant responses, likely through Nrf2 pathway activation, leading to the upregulation of SOD, a key enzyme involved in neutralizing ROS. Although ivy and heather honey exhibited lower ORAC and DPPH values compared to those of manuka honey, their stronger induction of Nrf2 suggests that their bioactive compounds may stimulate antioxidant defence mechanisms through pathways beyond direct radical scavenging. These effects may be mediated by unique phenolic compounds or other bioactive constituents that selectively activate intracellular redox signalling.

## 3. Discussion

This study investigated the antioxidant and immunomodulatory properties of Irish ivy and heather honey in comparison to those of manuka honey using PMA-differentiated THP-1 M1 macrophages as an exposure system. By assessing antioxidant/radical scavenging capacity (TPC, DPPH, and ORAC), cytotoxicity, pro- and anti- inflammatory gene expression (NF-κB, TNF-α, IL-1β, and IL-10), and the biomarkers of antioxidant response (Nrf2 and SOD), we sought to determine whether these Irish monofloral honey varieties possess therapeutic potential. The findings highlight distinct differences in the phenolic content and radical scavenging abilities, as well as variations in the capacity to modulate inflammation and oxidative stress in macrophages. Notably, ivy and heather honey demonstrated distinct bioactive properties, with ivy honey primarily stimulating the pro-inflammatory pathways through NF-κB and TNF-α activation (Figure 5A,B). Heather honey exhibited a dual effect by promoting both the pro-inflammatory (NF-κB and IL-1β) and anti-inflammatory (IL-10) cytokines (Figure 5A,C,D), respectively. Additionally, both the honey varieties significantly upregulated the antioxidant transcription factor Nrf2 and its downstream enzyme SOD, suggesting a strong capacity for oxidative stress regulation (Figure 6). These findings highlight the potential of ivy and heather honey as natural immunomodulatory and antioxidant agents, with possible applications in redox homeostasis and inflammation management. Overall, the implications of these findings are discussed in the context of existing research, with a focus on how these honey varieties compare to the established medicinal honey and their possible applications in inflammation management and wound healing.

### 3.1. Radical Scavenging Potential

The antioxidant potential of Irish ivy and heather honey evaluated through the TPC, DPPH radical scavenging, and ORAC assays, highlights their bioactive potential. The high TPC values of heather honey (104.8 mg/100 g) in this study, which were greater than those of manuka (93.86 ± 25.28 GAE mg/100 g honey) (Figure 1), align with those reported for other heather honey varieties, such as those from the Western Balkans (79.3–84.0 mg/100 g) [22], Spain (163 mg/100 g) [42], and Poland (125.8–195.1 mg/100 g) [19]. While the TPC for Irish ivy honey (50.37 mg/100 g) was found to be lower than those of both the heather and manuka honey (Figure 1), this result for Irish ivy honey aligns with prior research by Kolaylı et al. [28], which reported a TPC of 45.23 mg/100 g for Turkish ivy honey.

The DPPH RSA results were consistent with those of prior studies, although care must be taken with direct comparisons due to the differences in incubation times, solvent extractions, and concentrations tested [41,43,44]. In this study, methanolic extracts of manuka honey, 0.2 g/mL, scavenged 70.72% DPPH within 30 min, while ivy and heather honey scavenged 32.34% and 31.08%, respectively (Figure 2). This trend contrasts with the TPC results shown in Figure 1, where heather honey exhibited a higher TPC than manuka honey. These findings suggest that antioxidant capacity, as measured by DPPH radical scavenging, may not correlate directly with the TPC alone, indicating the potential involvement of additional non-phenolic compounds in manuka honey or differences in phenolic extractability and activity.

The differences in the bioactive constituents of Irish heather honey versus manuka honey may lead to a greater radical scavenging effect for the methanolic extracts of manuka honey. The choice of solvent has been shown to influence the DPPH results, with ethanol and water extractions yielding different outcomes [45]. Both heather and ivy honey achieved a comparable level of DPPH scavenging within a shorter incubation time, matching 36.77–42.37% scavenging observed in Belgian polyfloral honey after one hour of incubation at 0.2 g/mL [43]. Furthermore, Broznić et al. [44] reported that a 5% *w*/*v* aqueous honey solution scavenged 28–47% DPPH after one hour. Additionally, Ndungu et al. [41] reported 43.48% DPPH RSA for a 0.1 g/mL methanolic extract of UMF5+ manuka honey (85 mg/kg MGO) after fifteen minutes, while the other honey samples tested, such as unidentified Chogoria honey, showed RSA values as low as 8.45%. Future studies incorporating multiple solvent extractions should provide a more comprehensive assessment of Irish honey’s radical scavenging potential.

The ORAC assay further confirmed the antioxidant effects of ivy and heather honey. While the TE for Irish ivy honey (90.83 µM/g), was greater than that of heather honey (71.46 µM/g), those of both the honey samples were lower than that of manuka honey (150.39 µM/g) (Figure 3). These findings were higher than that of stingless bee honey (1.99–6.67 µM/g) [46]. Overall, the ORAC values were also notably higher than those of the Hungarian honey varieties, such as linden, acacia, and chestnut (13.79–114.89 µM TE/g) [47,48]. The strong ORAC (peroxyl radical neutralising) values for Irish ivy and heather honey support their potential role in oxidative stress regulation by enhancing the cellular defence mechanisms against ROS.

A correlation between the ORAC values and other bioactive properties has been suggested in previous studies [11], supporting the idea that high antioxidant capacity may contribute to honey’s broader therapeutic potential. While the relationship between antioxidant activity and antimicrobial properties is not yet fully understood, previous research has indicated that phenolic compounds and antioxidant mechanisms may play a role in influencing microbial environments [49,50]. The variation in the ORAC values for manuka honey across studies, such as those reported by Marshall et al. [51] (6.92–11.3 µM TE/g), highlights the influence of the extraction techniques, the honey dilution factors, and botanical variability. The antioxidant potential of Irish ivy and heather honey is supported by their TPC, DPPH radical scavenging ability, and ORAC values, suggesting that the bioactive compounds present in these Irish honey samples contribute not only to free radical neutralisation, but also to broader oxidative stress regulation, reinforcing their therapeutic potential in immune modulation and cellular protection.

### 3.2. Changes in Inflammatory and Antioxidant Marker Gene Expression by Irish Honey Varieties

Honey’s immunomodulatory and antioxidant properties have gained increasing attention due to their potential therapeutic applications in inflammation management and oxidative stress regulation. In this study, ivy and heather honey were evaluated for their ability to modulate immune responses and enhance antioxidant defences in PMA-differentiated THP-1 M1 macrophages using manuka honey as a standardised medicinal honey comparator. Gene expression analysis revealed that both the Irish honey varieties exhibited distinct immunomodulatory and antioxidant properties, influencing key pro- and anti-inflammatory mediators, such as NF-κB, TNF-α, IL-1β, and IL-10, as well as the antioxidant markers Nrf2 and SOD. These findings contribute novel insights into the bioactivity of Irish monofloral honey, reinforcing its potential therapeutic relevance in inflammation-related conditions.

#### 3.2.1. Resazurin-Based Cytotoxicity Evaluation of Irish Honey

A preliminary cytotoxicity assessment was conducted to establish sub-cytotoxic concentrations for subsequent gene expression analyses, ensuring that any observed immunomodulatory or antioxidant effects were not a consequence of cellular toxicity.

All the three honey samples (ivy, heather, and manuka) exhibited a dose-dependent cytotoxic effect, with higher concentrations (≥10% *v*/*v*) significantly reducing THP-1 macrophage viability after 12 and 24 h (Figure 4A,B).

At 20% (*v*/*v*) for 24 h, cell viability decreased for ivy (2.95% ± 2.526), heather (2.497% ± 0.698), and manuka honey (2.312% ± 0.809), with ivy honey being the least cytotoxic (Figure 4A). At 20% (*v*/*v*) for 12 h, ivy honey resulted in 27.97% ± 8.11 viability compared to those of heather (5.17% ± 2.91, *p* < 0.01) and manuka (8.03% ± 8.52, *p* < 0.05) (Figure 4B). After 6 h at 20% (*v*/*v*), cell viability again remained higher for ivy honey (55.34% ± 16.4) compared to those of heather (34.65% ± 4.23) and manuka (26.13% ± 6.95) (Figure 4C).

These findings suggest that ivy honey is the least cytotoxic, particularly at shorter exposure times, while heather and manuka honey exert stronger cytotoxic effects at higher concentrations and longer durations. The observed differences in cytotoxicity may be attributed to the bioactive compounds present in each honey type, warranting further investigation. Manuka honey, rich in MGO (~25 mg/100 g), has been reported to exhibit cytotoxic effects (EC_50_ 21.6 mg/100 mL [52,53,54]) due to its role in advanced glycation end-product (AGE) formation [52,53,54,55].

Comparing these results with those of previous studies, Afrin et al. [56] observed 100% viability in RAW 246.7 macrophages for 0.1–1% (*w*/*v*) manuka honey after 24 h, whereas Ooi et al. [57] reported a 20% reduction in viability in RAW 246.7 macrophages for 2.5% (*v*/*v*) stingless bee honey after 24 h. In the present study, after 24 h, a slight reduction was observed at 5% (*v*/*v*), but viability was still above 80% for ivy (87.95% ± 3.89), heather (82.68% ± 4.92), and manuka (80.34% ± 5.17) (Figure 4A). Cell viability remained at 100% when the cells were exposed to 2.5% (*v*/*v*) ivy, heather, or manuka honey for 24, 12, or 6 h (Figure 4A–C). These findings align with those of previous studies by confirming that 2.5% (*v*/*v*) honey is a sub-cytotoxic concentration, with only minor reductions in cell viability across all the tested honey samples at 5% (*v*/*v*), similar to the 20% viability reduction reported for stingless bee honey at 2.5% (*v*/*v*) in RAW 264.7 macrophages [57]. This concentration is therefore suitable for investigating the immunomodulatory and antioxidant responses in macrophages without inducing significant cytotoxic effects.

#### 3.2.2. The Effects of Irish Honey Varieties on Pro- and Anti-Inflammatory Marker Gene Expression

To assess the immunomodulatory potential of ivy and heather honey, the expression of the key inflammatory biomarkers NF-κB, TNF-α, IL-1β, and IL-10 was evaluated in the LPS-stimulated THP-1 macrophages following 6 h exposure to 2.5% (*v*/*v*) honey. All the gene expression values were normalised to the housekeeping genes RPL37A and β-actin using the ΔΔCt method, with the results expressed relative to the LPS-stimulated controls (Figure 5).

NF-κB Expression

NF-κB is a central transcriptional regulator of inflammation and immune activation, primarily driving the expression of pro-inflammatory cytokines, such as TNF-α and IL-1β [58]. Both ivy (2.9-fold ± 0.67, *p* < 0.001) and heather honey (3.985-fold ± 0.11, *p* < 0.0001), significantly upregulated NF-κB expression relative to that of the LPS-treated control cells, with heather honey exerting the greatest effect (Figure 5A). Manuka honey also increased NF-κB expression (2.268-fold ± 0.545, *p* < 0.05), with the lab honey having the smallest effect of all the honey samples tested (1.322-fold ± 0.31).

These findings align with Raynaud et al. [59], who reported stimulation in NF-κB in RAW 246.7 macrophages exposed to thyme honey. The strong NF-κB induction by both the ivy and heather honey suggests they may contain similar bioactive components, warranting further investigation.

TNF-α Expression

TNF-α is a key pro-inflammatory cytokine regulated by NF-κB, playing a pivotal role in the early immune response [60]. Ivy (3.23-fold ± 1.22, *p* < 0.05) and heather honey (2.397-fold ± 0.608) both significantly increased TNF-α expression, as did manuka honey (2.795-fold ± 0.87, *p* < 0.05), with ivy honey exerting the highest level of TNF-α induction among the honey samples tested (Figure 5B). The lab honey induced only a slight increase in TNF-α expression normalised to control gene expression in the LPS-stimulated macrophages (1.322-fold ± 0.312) (Figure 5B).

The similarity between ivy and manuka honey in TNF-α upregulation suggests they may share similar immune-stimulatory mechanisms, possibly involving TLR4-NF-κB signalling. Tonks et al. [61], identified a 5.8 kDa bioactive component in manuka honey that activated NF-κB via TLR4, leading to pro-inflammatory TNF-α and IL-1β production [61]. Gannabathula et al. [36] reported that arabinogalactan from kanuka honey stimulated TNF-α via NF-κB activation in THP-1 cells [36]. Duncan et al. [62] observed an increase in TNF-α in differentiated THP-1 macrophages also stimulated with LPS and pretreated with clover honey gold nanoparticles (AuNP). Alvarez-Suarez et al. [63] hypothesised that manuka honey activates TNF-α via the Nrf2-MAPK pathway, a mechanism that may also apply to ivy honey, given the similarities in their NF-κB-driven cytokine activation [56,63].

IL-1β Expression

Pro-IL-1β is active by the NF-κB pathway. Heather honey exhibited the strongest IL-1β induction (1.63-fold ± 0.17, *p* < 0.01), whereas manuka (1.267-fold ± 0.17), ivy (1.17-fold ± 0.16), and the lab honey (1.211-fold ± 0.29) caused only minor changes (Figure 5C). The significant upregulation of IL-1β by heather honey is in line with its upregulation of NF-κB (Figure 5A). IL-1β stimulation has been observed with manuka honey [64], acacia honey [34], clover honey AuNP [62], and alhagi honey polysaccharides [65]

IL-10 Expression

IL-10 is an anti-inflammatory cytokine that plays a crucial role in immune resolution by counteracting NF-κB-driven inflammation [66]. Heather honey induced the highest IL-10 expression (1.757-fold ± 0.78), while ivy honey caused only a slight increase (1.086-fold ± 0.1713) (Figure 5D). In contrast, manuka honey (0.852-fold ± 0.29) and the lab honey (0.856-fold ± 0.62) suppressed IL-10 expression more compared to that of the LPS stimulated macrophage control.

This finding suggests that heather honey exerts immunoregulatory effects, balancing the pro-inflammatory (NF-κB and IL-1β) and anti-inflammatory (IL-10) cytokines, similar to findings from Harakeh et al. [67], who reported honey-induced IL-10 production, while modulating NF-κB activity [67]. This may suggest that heather honey may modulate immune responses through more than one inflammatory pathway. The minor IL-10 response in the ivy honey-treated cells further supports its role as a pro-inflammatory immune activator rather than an immunomodulator (Figure 5D).

Similar to thyme honey [59], ivy honey upregulated NF-κB, TNF-α, and IL-1β, suggesting a role in immune activation (Figure 5A–C). Heather honey’s ability to stimulate both the pro-inflammatory (TNF-α and IL-1β) and anti-inflammatory (IL-10) cytokines (Figure 5B–D) mirrors the findings on manuka honey [68], with an increasing number of pro-inflammatory cytokines (TNF-α and IL-1β) and an increase in the IL-10 level [37], which have been shown to both activate and regulate immune responses. In contrast, some honey varieties, such as Brazilian honey [69] and chestnut honey [70], have demonstrated the strong suppression of NF-κB and pro-inflammatory cytokines. However interestingly in this study, both ivy and heather honey appear to act as both pro- and anti-inflammatory immune modulators (Figure 5A–D).

As expected, the lab honey had minimal immunomodulatory effects, reinforcing the hypothesis that bioactive compounds, rather than sugar content, drive the immune response.

#### 3.2.3. Effects of Irish Honey Varieties on Antioxidant Transcription Factor and Associated Enzyme Expression

To assess the antioxidant potential of ivy and heather honey, the expression of Nrf2, a transcriptional regulator of cellular antioxidant defences, and SOD, a key enzyme involved in ROS detoxification, was evaluated in the LPS-stimulated THP-1 macrophages following 6 h exposure to 2.5% (*v*/*v*) honey. All the changes in the gene expression values were normalised to the housekeeping genes RPL37A and β-actin, with the results expressed relative to the LPS-stimulated control cells (Figure 6).

Nrf2 Expression

Nrf2 is a master regulator of cellular antioxidant defence, activating the genes responsible for mitigating oxidative stress [71]. Ivy honey induced the highest upregulation of Nrf2 (3.287-fold ± 0.159, *p* < 0.001), followed by heather honey (2.533-fold ± 0.743, *p* < 0.01), and then the LPS-stimulated control (Figure 6A). Manuka honey induced a moderate increase in Nrf2 (1.598-fold ± 0.42), whereas the lab honey had a minimal effect (1.173-fold ± 0.109).

These findings indicate that ivy honey is a strong activator of Nrf2, suggesting that it has an ability to stimulate cellular antioxidant responses. The potential activation of Nrf2 by ivy and heather honey is shown in Figure 6A. Since Nrf2 activation is often associated with phenolic compounds [72], these results suggest a possible link between the honey polyphenol content and the Nrf2-mediated antioxidant responses. However, Figure 1 indicates that heather honey has a higher TPC than manuka honey, while Figure 6A shows that ivy honey, rather than heather, has the greatest effect on Nrf2 activation. Ivy honey may contain bioactive compounds that although less effective in direct radical neutralisation, stimulate intracellular antioxidant pathways more effectively than manuka honey. Additionally, differences in phenolic composition, solubility, or the cellular uptake of honey-derived compounds could influence the activation of Nrf2, despite the lower ORAC/DPPH values. Further studies on the specific phenolic profiles and the cellular uptake mechanisms of ivy honey are needed to clarify this distinction.

These findings align with previous research, demonstrating that manuka honey upregulates Nrf2 in human dermal fibroblasts [63] and RAW 264.7 macrophages [37]. Some studies on stingless bee honey [73] and bitter gourd honey [74] have also shown that honey can activate Nrf2 and other antioxidant pathways in models of inflammation and diabetes. However, the higher-level induction of Nrf2 by ivy and heather honey compared to that of manuka honey, Figure 6, suggests that these Irish honey varieties may offer enhanced antioxidant protection, warranting further investigation.

SOD Expression

SOD is a crucial enzyme in neutralising superoxide radicals, thereby preventing oxidative damage [75]. Heather honey induced the highest upregulation of SOD (4.95-fold ± 1.69, *p* < 0.01), followed closely by ivy honey (4.68-fold ± 1.69, *p* < 0.01) (Figure 6B). Manuka honey also increased SOD expression, but to a lesser extent (2.521-fold ± 0.811, *p* < 0.05), while the lab honey induced a minimal effect (1.565-fold ± 0.596).

Although ivy honey induced the highest Nrf2 expression, both ivy and heather honey upregulated SOD to a similar extent, Figure 6B, suggesting that these honey samples can enhance antioxidant defences through both transcriptional regulation (Nrf2) and enzymatic activity (SOD). Given that SOD is a direct target of Nrf2, the strong induction of both these genes supports the hypothesis that ivy and heather honey activate antioxidant pathways in a coordinated manner (Figure 6).

Notably, the higher SOD expression observed with ivy and heather honey compared to that of manuka honey suggests a stronger ability to enhance ROS detoxification, further reinforcing their antioxidant effects. This is consistent with the previous studies demonstrating that honey-derived bioactive compounds enhance SOD activity in oxidative stress models [76].

Interestingly, not all the studies report the uniform upregulation of antioxidant responses with honey treatment. Afrin et al. [56] found that manuka honey suppressed Nrf2, SOD, and other antioxidant enzymes (CAT, GPx, GR, and HO-1) in HCT-116 and LoVo cancer cell lines, suggesting that honey’s effects may vary depending on the cell type and the metabolic state. This highlights the complexity of honey’s bioactivity and suggests that its antioxidant properties may be context-dependent, with beneficial effects in normal and inflamed cells, but potential suppression in malignancies.

The mechanism underlying the antioxidant effects of honey may involve the phenolic compounds and polysaccharides present in the honey samples, which could activate Nrf2. Some studies have shown that the polysaccharides from medicinal plants such as *Polygonatum cyrtonema* enhance SOD and Nrf2 expression in LPS-induced acute lung injury models [76], while honey-derived polysaccharides have been linked to reduced IL-1β, IL-6, and TNF-α levels, alongside increases in IL-10 and Nrf2 activation [77]. This suggests that specific honey-derived bioactive components may be responsible for triggering the Nrf2 pathways, providing a mechanistic basis for the observed increase in antioxidant markers.

Furthermore, the findings from various in vivo animal studies further support the role of honey in stimulating antioxidant defences. Harakeh et al. [67] demonstrated that wadi and talh honey increased SOD and CAT expression in indomethacin-induced gastric ulcer models, while Malkoç et al. [78] observed that rhododendron honey enhanced wound healing in diabetic rats by upregulating GPx, SOD, and CAT expression and reducing the TNF-α levels. Abouzed et al. [79] also reported similar effects with Egyptian honey, showing increased antioxidant enzyme activity and reduced oxidative stress markers in hepatocellular carcinoma models. These studies provide strong in vivo evidence that honey enhances cellular antioxidant defences, reinforcing its therapeutic potential in oxidative stress-related conditions.

The strong induction of Nrf2 and SOD by ivy and heather honey in this study may correlate with their TPC and RSA (Figure 1, Figure 2 and Figure 3) [13,72]. Phenolic compounds are well-documented Nrf2 activators [72]; it may be that their presence in in ivy and heather honey (Figure 1) likely contributed to the greater upregulation of antioxidant genes compared to that of manuka honey (Figure 6). However, despite their lower ORAC and DPPH values relative to those of manuka honey, ivy and heather honey may contain other bioactive compounds that selectively activate Nrf2 and SOD through alternative pathways separate from direct radical scavenging. These mechanisms could involve polyphenols, flavonoids, or bioactive compounds such as polysaccharides [65] that interact with cellular redox sensors or modulate intracellular antioxidant responses beyond their immediate ROS neutralization capacity. Further studies are required to identify these compounds and elucidate their specific roles in triggering antioxidant pathways.

Future research should explore whether these effects translate into long-term protection against inflammation-induced oxidative damage and whether honey treatment could modulate macrophage polarization toward an M2 anti-inflammatory state.

## 4. Materials and Methods

### 4.1. Materials

RPMI 1640 media, foetal bovine serum, L-glutamine, penicillin–streptomycin, trypan blue, Dulbecco’s phosphate-buffered saline (without CaCl_2_ and MgCl_2_), sodium carbonate, Qubit RNA high-sensitivity assay kit, Superscript III Reverse Transcriptase, Oligo (dT)_18_, and RNaseOut were purchased from Thermo Fisher Scientific Dublin, Ireland. PMA (P8138) and LPS (L4391), Folin’s solution, gallic acid, Whatman No. 1 filter paper sucrose, maltose, glucose, fructose, methanol, DPPH, Trolox, sodium fluorescein, APPH oxidant (2, 20-azobis (2-amidinopropane) dihydrochloride), KH_2_PO_4_, K_2_HPO_4_, Roche High Pure RNA Isolation kit, and resazurin sodium salt were all purchased from Sigma-Aldrich (Merck Life Science Ltd., Wicklow, Ireland). Meridian dNTP mix and 0.2 μM PES syringe filters were purchased from Scientific Laboratory Supplies, Dublin, Ireland.

### 4.2. Honey Samples

Unpasteurised Irish honey samples were sourced in 2021 directly from local beekeepers, with the assistance of the Native Irish Honey Bee Society (NIHBS), the Federation of Irish Beekeepers’ Association (FIBKA), and the Irish Beekeeping Association (IBA). The monofloral status of the ivy and heather honeys was confirmed by melissopalynological analysis [80,81,82], supported by the expert verification of pollen types at the University of Galway (see Supplementary Method S1 and Supplementary Table S1). The heather honey samples contained over 70% *Calluna vulgaris* pollen, while the ivy honey samples contained over 80% *Hedera helix* pollen. All the honey samples were screened for bacterial and fungal contamination prior to use (Supplementary Method S2), and only uncontaminated samples were included in antioxidant and immunomodulatory testing.

A single batch of medical-grade, quality-assured manuka honey (MGO 250+; Manuka Health, Wicklow, Ireland) was used as a comparator. Additionally, laboratory-formulated honey (lab honey) was prepared to serve as a sugar control [31,83]. The lab honey consisted of 1.5 g sucrose, 7.5 g maltose, 40.5 g fructose, and 33.5 g glucose (Merck, Dublin, Ireland) dissolved in 17 mL of sterile distilled water to create a 50% (*w*/*v*) stock solution [83]. This formulation was based on previous studies that have used similar artificial sugar mixtures as negative controls to account for osmotic effects [31,83]. Moreover, the sugar composition aligns with reported averages for natural honeys across various floral and geographical sources [84,85]. This stock was subsequently diluted to match the final concentrations used for the natural honey samples. All the honey samples were stored in the dark at 4 °C until use.

### 4.3. Antioxidant Assays

#### 4.3.1. Total Phenolic Content

The Folin–Ciocalteu assay was conducted following the method of Singleton et al. [86]. In brief, a 7.5 g/100 mL sodium carbonate solution (Thermo Scientific, Dublin, Ireland), a 0.2 N Folin’s solution (Merck, Dublin, Ireland), and standard dilutions of gallic acid in water (200 mg/L, 180 mg/L, 120 mg/L, 80 mg/L, 40 mg/L, and 0 mg/L) (Merck, Dublin, Ireland) were prepared. Honey was diluted in sterilised distilled water to create a 10% (*w*/*v*) solution, which was then filtered through Whatman No. 1 filter paper (Merck, Dublin, Ireland) to remove pollen grains and debris. Subsequently, 1 mL of each honey sample received, along with manuka honey, was incubated for 5 min with 5 mL of 0.2 N Folin’s solution. Following this, a 4 mL volume of the sodium carbonate solution (7.5 g/100 mL) was added to the mixture and incubated at room temperature in the dark for 1 h.

Absorbance readings for each honey sample were measured in triplicate at 760 nm using a UV-Vis spectrometer (Shimadzu 1800, UV-Vis spectrophotometer, Mason technology, Dublin, Ireland), with measurements taken against a 0 mg/L blank. The TPC was quantified based on a standard curve prepared with known concentrations of gallic acid. To ensure the reliability of the results, each experiment was performed independently on two separate occasions for all the honey samples. The TPC was then calculated and expressed as mg GAE/100 g of honey.

#### 4.3.2. Preparation of Honey Extracts for DPPH and ORAC Assays

For antioxidant capacity testing, methanolic extracts of the honey samples were prepared. A total of 2 g honey was added to a 15 mL falcon tube, followed by 10 mL of 99% methanol (Merck, Dublin). The mixture was vortexed followed by filtration using Whatman No. 1 filter paper (Merck, Dublin). Methanolic extracts were stored at −80 °C in a freezer until further testing.

#### 4.3.3. DPPH Assay

For the multiwell plate DPPH assay, we followed the method described by Ali et al. [87], with slight modifications. The concentration of DPPH was selected to achieve an absorbance of less than 1.0 at 517 nm, ensuring compliance with Beer–Lambert’s law. A volume of 140 μL of either the blank, methanolic honey solution (0.2 g/mL), or Trolox (80 μM) as a positive antioxidant control was added to a 96-well plate, followed by 40 μL of 1.5 mM DPPH (Merck, Ireland) in methanol. The plate was incubated in the dark at room temperature for 30 min. Absorbance was then measured at 517 nm with the Agilent Gen 5 Biotek reader, Mason technology, Dublin, Ireland. The percent of DPPH scavenged by honey was calculated using Equation (1).

(1) $$\%DPPH\;scavenged=\frac{ABS(sample+DPPH)}{ABS(untreated\;DPPH)}\times 100$$

#### 4.3.4. The ORAC Assay

The ORAC assay was performed following a protocol adapted from Farkas et al. [11], with specific modifications to optimise the conditions for this study. Working solutions of sodium fluorescein (4 nM) (Merck, Dublin) and the APPH oxidant (2, 20–azobis (2-amidinopropane) dihydrochloride (101.7 g in 5 mL) were prepared in 75 mM potassium phosphate buffer (KH_2_PO_4_ and K_2_HPO_4_ (Merck, Dublin)) at pH 7.5. A stock solution of Trolox (160 mM) was first prepared in methanol, and subsequent 1 mL working solutions (1:2 dilutions) were diluted in Eppendorf tubes with potassium phosphate buffer (0–160 μM) immediately before each experiment.

In each well of a 96-well plate, 25 μL of the blank (potassium phosphate buffer), the standard (Trolox 10–160 µM), and the samples (range of honey samples) were aliquoted along with 150 μL of the fluorescein working solution. The mixture was incubated in the dark at 37 °C for 30 min. After incubation, 25 μL of the AAPH oxidant solution was added to each well. The plate was then immediately placed in a pre-warmed Agilent Gen 5 Biotek reader (37 °C). The wells were shaken at maximum intensity for 10 s. Fluorescence readings were taken every minute, with an initial autoscale option for gain optimisation. The excitation and emission wavelengths were set at 485 nm and 528 nm, respectively.

The ORAC values were calculated using the area under the curve (AUC) (2) and the net AUC (3) of the standard and samples. The honey samples’ Trolox equivalence was interpolated from the Trolox standard curve.

(2) $$AUC=\left(\frac{R_{1}}{R_{1}}\right)+\left(\frac{R_{2}}{R_{1}}\right)+\left(\frac{R_{3}}{R_{1}}\right)+\left(\frac{R_{4}}{R_{1}}\right)+\ldots +\left(\frac{R_{n}}{R_{1}}\right)$$

where R_1_ is the fluorescence reading at the initiation of the reaction and R_n_ is the last measurement.

(3) $$Net\;AUC={AUC}_{sample}-{AUC}_{blank}$$

### 4.4. Immunomodulatory and Antioxidant Effects on Differentiated THP-1 Cells

Gene expression changes in the inflammatory and antioxidant biomarkers were analysed in the pro-inflammatory stimulated M1 macrophages exposed to sub-cytotoxic concentrations of the various honey varieties (heather and ivy), a standardised manuka honey control, and a sugar control (lab honey) to represent osmotic effects. The THP-1 cells were differentiated using 15 ng/mL PMA for 72 h, followed by a 24 h rest, and then stimulated with 0.1 µg/mL LPS for 24 h to achieve a pro-inflammatory state. The cells were subsequently treated with 2.5% (*v*/*v*) honey for 6 h, after which RNA was extracted, cDNA-synthesised, and qPCR was conducted to assess gene expression. Changes in the biomarkers, such as NF-κB, TNF-α, IL-1β (pro-inflammatory), IL-10 (anti-inflammatory), and Nrf2 and SOD (antioxidant), were analysed to evaluate the inflammatory and antioxidant effects of honey.

#### 4.4.1. Culture of THP-1 Cells

The THP-1 cells (ECACC 88081201) were maintained in a biobank at the Bioscience Research Institute. THP-1 cells are a human monocytic cell line widely used for gene expression and immunological analysis due to their resemblance to primary monocytes and their ability to differentiate into macrophages under specific conditions [88,89]. The THP-1 cells (ECACC 88081201) between passages 4 and 15 were cultured in RPMI 1640 medium supplemented with 10% foetal bovine serum, 1% L-glutamine, and 1% penicillin–streptomycin in a humidified incubator at 37 °C in 5% CO_2_. The cells were maintained at a cell density of 4 × 10^5^ cells/mL up to 1.6 × 10^6^ cells/mL, with media refreshed every four days and sub-cultured once every 10–14 days. The cells were seeded at various densities 18 h prior to PMA exposure for all the experiments to allow for cell recovery.

#### 4.4.2. Honey Cytotoxicity

The cytotoxicity of honey was evaluated using the resazurin assay with certain modifications [90]. The THP-1 cells were seeded at 5 × 10^4^ cells/100 µL/well in 96-well plates and differentiated according to the optimised PMA protocol (15 ng/mL for 72 h followed by 24 h rest in PMA free media) [38]. A stock concentration of honey was filter-sterilised using a 0.2 µm polyethersulfone (PES) syringe filter from which working solutions were prepared by dilution in complete cell culture media to final concentrations of 20%, 10%, 5%, and 2.5% (*v*/*v*), which were added to the wells (100 µL/well), and the plates were incubated for a further 6, 12, or 24 h at 37 °C in a 5% CO_2_ incubator. Prior to the addition of 440 µM stock solution of resazurin (44 µM final concentration per well) and further incubation at 37 °C for 3 h, the honey was removed, and the plates were washed three times with Dulbecco’s PBS (without calcium or magnesium) to prevent the interference of honey with the resazurin assay [91]. Relative fluorescence units were measured at Ex 528 nm and Em 590 nm with the Agilent Gen 5 Biotek reader.

#### 4.4.3. Gene Expression Analysis of M1 THP-1 Macrophages Exposed to Honey

The cells were seeded at 1 × 10^6^ cells/mL in a six-well plate. The THP-1 cells were cultured overnight, and then treated as per the optimised PMA protocol (15 ng/mL for 72 h followed by 24 h rest in PMA free media) [38]. After the 24 h rest period, macrophages were exposed to 0.1 ug/mL of LPS for 24 h to polarise the macrophages to an M1 state. Honey, after filter sterilisation (using a 0.2 μM PES syringe filter), was added to the wells at a final sub-cytotoxic concentration of 2.5% (*v*/*v*) for 6 h. Three independent experiments were conducted for each treatment type (i.e., LPS only, ivy honey, heather honey, manuka honey, and lab honey).

The cells were washed with 2 mL PBS and harvested by scraping, followed by centrifugation to pellet the cells at 300× *g* for 5 min. The pellet was resuspended in 200 μL of PBS as per the manufacturer’s guideline for Total RNA extraction. The Roche High Pure RNA Isolation Kit (Merck, Dublin, Ireland) was used to extract Total RNA as per the manufacturer’s guidelines. RNA was eluted in 50 μL of elution buffer and stored at −80 °C until further processing.

The quality of RNA samples was assessed using the NanoDrop Lite Plus (Thermo Scientific, Dublin, Ireland) to check for DNA/protein (A_260_/A_280_) and/or other contaminants, such as organic compounds, salts, and carbohydrates (A_260_/A_230_), and the Qubit 3.0 (Thermofisher, Dublin, Ireland) was used to assess RNA quantity (ng/µL) as per the manufacturer’s guidelines.

The synthesis of cDNA was carried out using Superscipt III Reverse Transcriptase (Invitrogen, Dublin, Ireland) using 100 ng RNA per reaction and the Applied Biosystems MiniAmp thermocycler as per the manufacturer’s guidelines. A volume of 1 µL Oligo (dT)_18_ primer (Invitrogen, Ireland), 1 µL 10 mM dNTP mix (Meridian, Scientific Laboratory Solutions, Dublin, Ireland), RNA (100 ng), and RNAse-free water up to 13 µL was added to a tube and heated to 65 °C for 5 min. After this, 5 µL 5× First-Strand buffer, 1 µL 0.1 M DTT, 1 µL Superscript III RT, and 1 µL RNaseOut (Invitrogen, Ireland) were added to the reaction tube. The tubes were further incubated at 50 °C for 60 min, followed by inactivating the reaction by heating to 70 °C for 15 min. All the samples were stored at −80 °C until further testing.

The qPCR reactions were prepared in a total volume of 10 µL, consisting of 1X PowerUp SYBR Green Master Mix (Applied Biosystems, ThermoScientific, Dublin, Ireland), 0.4 µM forward primer and 0.4 µM reverse primers (primer sequences detailed in Table 1), 1 µL of cDNA template (equivalent to 100 ng of input RNA), and 3.2 µL of nuclease-free water.

Thermal cycling was performed using the Roche LightCycler^®^ 96 with the following conditions: initial pre-incubation at 50 °C for 120 s, followed by denaturation at 95 °C for 120 s. This was succeeded by 40 cycles of two-step amplification, comprising denaturation at 95 °C for 15 s and annealing/extension at 60 °C for 60 s, with fluorescence data acquisition at the annealing/extension step. Post-amplification, melting curve analysis was conducted to verify the specificity of the amplification products. This included denaturation at 95 °C for 15 s, cooling to 60 °C for 60 s, and a final temperature ramp to 95 °C for 15 s with 15 readings/°C.

As per the MIQE guidelines, the PCR primers were optimised prior to the final experiment. Primer efficiency was evaluated using a 1:10 serial dilution of cDNA, ensuring efficiencies between 90 and 110%, with an R^2^ value > 0.99. Reference gene suitability was assessed by testing three candidates—RPL37A, β- actin, and GAPDH [92]—across the various treatment conditions to confirm uniform expression independent of the experimental conditions.

**Table 1:** Primer sequences used for qPCR gene expression analysis of honey-treated cells.

Gene	Sequence (5′-3′)	Reference
**GAPDH**	F-GGTGGTCTCCTCTGACTTCAACA	[93]
	R-GTTGCTG TAGCCAAATTCGTTGC	
**RPL37A**	F-ATTGAAATCAGCCAGCACGC	[92]
	R-AGGAACCACAGTGCCAGATCC	
**Β-actin**	F-ATTGCCGACAGGATGCAGAA	[92]
	R-GCTGATCCACATCTGCTGGAA	
**NF-κB**	F-TGAGTCCTGCTCCTTCCA	[94]
	R-GCTTCGGTGTAGCCCATT	
**TNF-α**	F-CCTCTCTCTAATCAGCCCTCTG	[95]
	R-GAGGACCTGGGAGTAGATGAG	
**IL-1β**	F-ATGATGGCTTATTACAGTGGCAA	[93]
	R-GTCGGAGATTCGTAGCTGGA	
**IL-10**	F-GACTTTAAGGGTTACCTGGGTTG	[93]
	R-TCACATGCGCCTTGATGTCT	
**Nrf2**	F-CTTGGCCTCAGTGATTCTGAAGTG	[96]
	R-CCTGAGATGGTGACAAGGGTTGTA	
**Mn-SOD**	F-GGGAGCACGCTTACTACCTTC	[97]
	R-TCTTGCTGGGATCATTAGGGTAT	

The geometric mean of two of the reference genes (RPL37A and βactin) was calculated, and delta delta PCR was used to calculate the relative fold change in treatments to the LPS-treated control (4)–(6).

(4) dCq control=Target gene of control−Reference gene of control

(5) dCq sample=Target gene of sample−Reference gene of sample

(6) ddCq=dCq sample−dCq control

### 4.5. Statistical Analysis

All the experiments included a minimum of three replicates, and three independent experiments unless stated otherwise. The data are reported as the mean ± standard deviation (std. dev.) unless otherwise stated in the figures. Statistical analysis included one-way ANOVA, followed by Bonferroni’s post hoc test and two-way ANOVA, followed by Tukey’s or Dunnett’s post-hoc tests. Significance was defined as *p* < 0.05. Statistical testing was performed using GraphPad Prism (version 8).

## 5. Conclusions

This study provides the first direct evidence that Irish ivy and heather honey exhibit distinct immunomodulatory effects on LPS-stimulated THP-1 macrophages, demonstrating both pro- and anti-inflammatory potentials. Ivy honey significantly upregulated NF-κB, TNF-α, and IL-1β, suggesting an immune-stimulatory effect, whereas heather honey promoted both the pro-inflammatory (NF-κB, TNF-α and IL-1β) and anti-inflammatory (IL-10) mediators, highlighting its role in immune regulation. These findings suggest that honey’s immunological impact may be dependent on the floral source, warranting further mechanistic studies to determine whether NF-κB activation leads to compensatory antioxidant responses, such as Nrf2 activation.

While manuka honey exhibited the highest radical scavenging capacity, the ivy and heather honey varieties significantly stimulated Nrf2 and SOD expression, which may suggest distinct antioxidant activation pathways. These findings highlight the potential antioxidant properties of the Irish honey varieties, likely mediated through both direct radical scavenging and the activation of endogenous antioxidant defences via Nrf2. This reinforces the importance of polyphenols and flavonoids in driving honey’s bioactivity, highlighting the need for future research to identify the key bioactive components, including arabinogalactans, defensin-1, and MRJPs, to better understand their role in immune modulation and oxidative stress regulation.

Collectively, these findings suggest the Irish ivy and heather honey varieties contain bioactive properties with potential antioxidant and immunomodulatory effects. Further studies are required to determine their clinical relevance and therapeutic applications. As the demand for natural and functional foods grows, these honey varieties represent valuable candidates for medicinal and nutraceutical applications, supporting their potential therapeutic integration into health interventions.

## Data Availability

The data presented in this study are available on request from the corresponding author until such time as the data can be uploaded to an open science repository. They will then be linked to the corresponding author’s ORCID. The data are not currently publicly available due to limited access.

## Supplementary Materials

The following supporting information can be downloaded at: https://www.mdpi.com/article/10.3390/ijms26083625/s1.

## Abbreviations

The following abbreviations are used in this manuscript:

AAPH	2,2′-Azobis(2-amidinopropane) dihydrochloride
AGE	Advanced glycation end-product
AMPK	AMP-activated Protein Kinase
AUC	Area under the curve
CD14	Cluster of Differentiation 14
DPPH	2,2-Diphenyl-1-Picrylhydrazyl
DPPH RSA	DPPH radical scavenging activity
EC50	Half-Maximal Effective Concentration
MAPK	Mitogen-Activated Protein Kinase
MGO	Methylglyoxal
MMP	Matrix Metalloproteinase
MRJP	Major royal jelly protein
NO	Nitric Oxide
ORAC	Oxygen Radical Absorbance Capacity
ROS	Reactive Oxygen Species
TLR	Toll-like Receptor
TPC	Total phenolic content
TE	Trolox equivalent
GAE	Gallic Acid Equivalent
