# Supplementary material for: The In Vitro Antioxidant and Immunomodulatory Effects of the Irish Monofloral Ivy and Heather Honey Varieties
Source: Int J Mol Sci. 2025 Apr 11;26(8):3625. doi: 10.3390/ijms26083625 (PMC12027192; doi:10.3390/ijms26083625)
Supplement: Supplementary file 1 [file ijms-26-03625-s001.zip › ijms-3564708-supplementary.pdf]

Supplementary Methods

Supplementary Method S1. Melissopalynological Analysis

A comprehensive melissopalynological analysis was conducted based on the established method of Louveaux, *et al.*, (1978) [80], with specific adaptations to optimise the accuracy of pollen identification. A sample size of 10 g of honey was selected to ensure recovery and counting of at least 200 pollen grains, providing a representative assessment of each honey's botanical composition.

Initially, 10 g of honey was mixed with 20 mL of warm distilled water in a centrifuge tube and thoroughly homogenised. The mixture was centrifuged at 3,000 rpm for 5 minutes, and the supernatant was discarded. This centrifugation step was repeated to remove soluble sugars and impurities. The resulting pellet was resuspended in distilled water, transferred to a glass centrifuge tube, and centrifuged again under the same conditions. The supernatant was decanted, and glacial acetic acid (Merck, Dublin, Ireland) was added to dehydrate the sample.

Following another centrifugation and decanting step, the sample was acetylated using a 9:1 mixture of acetic anhydride and concentrated sulfuric acid (Merck, Dublin, Ireland). The acetylation process involved gentle stirring and incubation in a 37 °C water bath for 180 seconds. The sample was then centrifuged again, and the acid was carefully removed. The pellet was washed twice with distilled water to neutralise residual acid and resuspended in glycerol for preservation.

Pollen identification was performed using the *Textbook of Pollen Analysis* [81] and the Global Pollen Project database [82], which classify pollen grains based on morphological features including aperture type, size, shape, exine texture, and surface ornamentation. If over 45% of the total pollen grains counted were from a single floral type (as per Louveaux *et al.*, 1978 [80]), the honey was classified as monofloral and attributed to that floral source.

Supplementary Method S2. Microbial Contamination Screening

To ensure sterility prior to cell-based assays, 0.2 g of each honey sample was inoculated into 10 mL of sterile tryptic soy broth (TSB) (Merck, Ireland) to detect bacterial contamination, and 10 mL of Sabouraud dextrose broth (SDB) (Merck, Ireland) to detect fungal contamination. Cultures were incubated at 37 °C (TSB) and 21 °C (SDB) for up to 16 days. Each culture was inspected daily for turbidity, indicating microbial growth. In cases where turbidity was observed, 100 μL of the broth was plated onto tryptic soy agar (for bacterial growth) or Sabouraud dextrose agar (for fungal growth) and incubated under the same conditions. This allowed visual confirmation of contamination. Samples that exhibited signs of contamination was excluded from subsequent immunological or viability experiments.

Supplementary Tables

Supplementary Table S1. Description of Honey Samples

Honey Type	Floral Source (Dominant Pollen)	Pollen Frequency (%)	Region Collected	Collection Year	Source	Confirmed by
Heather	*Calluna vulgaris*	≥70%	Leinster, Ireland	Late Sept 2021	Local beekeeper	Melissopalynology
Ivy	*Hedera helix*	≥80%	Leinster, Ireland	Nov 2021	Local beekeeper	Melissopalynology
Manuka	*Leptospermum scoparium*	Certified MGO 250+	New Zealand	[Batch Date]	Manuka Health	Commercial Certification
